# A Review on the Role of TRP Channels and Their Potential as Drug Targets_An Insight Into the TRP Channel Drug Discovery Methodologies

**DOI:** 10.3389/fphar.2022.914499

**Published:** 2022-05-24

**Authors:** Hamideh P Fallah, Ekta Ahuja, Haoquan Lin, Jinlong Qi, Qian He, Shan Gao, Hailong An, Jian Zhang, Yongzhen Xie, Dong Liang

**Affiliations:** ^1^ Aurora Biomed Inc., Vancouver, BC, Canada; ^2^ Department of Pharmacology, Hebei Medical University, Shijiazhuang, China; ^3^ Aurora Discovery Inc., Foshan, China; ^4^ Guangzhou Institute of Biomedicine and Health, Chinese Academy of Sciences, Guangzhou, China

**Keywords:** transient receptor potential (TRP) channels, ion channel reader (ICR), drug discovery, flux assay, non-radiolabeled ionic flux

## Abstract

Transient receptor potential (TRP) proteins are a large group of ion channels that control many physiological functions in our body. These channels are considered potential therapeutic drug targets for various diseases such as neurological disorders, cancers, cardiovascular disease, and many more. The Nobel Prize in Physiology/Medicine in the year 2021 was awarded to two scientists for the discovery of TRP and PIEZO ion channels. Improving our knowledge of technologies for their study is essential. In the present study, we reviewed the role of TRP channel types in the control of normal physiological functions as well as disease conditions. Also, we discussed the current and novel technologies that can be used to study these channels successfully. As such, Flux assays for detecting ionic flux through ion channels are among the core and widely used tools for screening drug compounds. Technologies based on these assays are available in fully automated high throughput set-ups and help detect changes in radiolabeled or non-radiolabeled ionic flux. Aurora’s Ion Channel Reader (ICR), which works based on label-free technology of flux assay, offers sensitive, accurate, and reproducible measurements to perform drug ranking matching with patch-clamp (gold standard) data. The non-radiolabeled trace-based flux assay coupled with the ICR detects changes in various ion types, including potassium, calcium, sodium, and chloride channels, by using appropriate tracer ions. This technology is now considered one of the very successful approaches for analyzing ion channel activity in modern drug discovery. It could be a successful approach for studying various ion channels and transporters, including the different members of the TRP family of ion channels.

## 1 Introduction

Transient receptor potential (TRP) channels are a large group of ion channels that have been predominantly found on the plasma membrane of various cell types (Himmel & Cox, 2020). These channels also have been found to be present on the organelles such as those in lysosomes and are known as organellar TRP channels (Zhang et al., 2018). These channels consist of several protein families and were initially found in a mutant strain of the fruit fly Drosophila (Cosens & Manning, 1969). So far, about 28 TRP channels have been discovered in various organisms that have been found to share some structural similarities (Islam, 2011). They are similar from their molecular sensing point as well as being non-selective cation channels with six transmembrane segments. However, these sub-families share little structural homology and are unique. These unique features lead to different sensory perception and regulation functions that TRP ion channels have throughout the body. TRP channels are categorized into two comprehensive groups known as group 1, including TRPV (vanilloid), TRPC (canonical), TRPM (melastatin), TRPA (ankyrin), and TRPN (no mechanoreceptor potential C) and group 2, including TRPP (polycystic) and TRPML (mucolipin). Also, an additional family known as TRPY has been discovered (Table 1). These channels are multifunctional signaling molecules that play various roles in cell physiology and sensory perception in health and disease conditions. These channels are located mainly on the surface of the cells as well as some organelles (Zhang et al., 2018). They are considered potential drug targets for various diseases such as neurological and psychiatric diseases, respiratory disorders, diabetes, as well as cancer. Remarkable is that the Nobel Prize in Physiology or Medicine in 2021 was awarded to David Julius and Ardem Patapoutian for the discovery of the key sensors of temperature and pressure, i.e., the thermally sensitive TRP channels and pressure-sensing Piezo channels (Earley et al., 2022). Noteworthy is that most of these TRP channels need intensive drug discovery and development efforts, and an improvement in technologies for these studies is necessary. In this review paper, we intend to discuss the most recent findings regarding the role of TRP ion channels as drug targets and also discuss the technologies that can improve the TRP ion channel drug discovery. We discuss that TRP channels respond to many types of different stimuli, the gating mechanism and ion flow are quite complex. However most of the known TRP channels are cation channels with some extend of action similarity. ICR has been shown to be a reliable instrument that can resolve many of the disadvantages of radiotracer assays for cationic channel activity by using non-radioactive ions as substitution for cations as tracers in bioassays. This instrument has various advantages such as increase in throughput, miniaturization, and reducing the signal-to-noise ratio using atomic absorption spectrometry combined with flux assay.

**TABLE 1:** Transient receptor potential channels (TRP ion channels) groups and subfamilies.

TRP ion channels superfamily							
Group 1			Group 2			Group TRPY	
G1	Subfamilies		G2	Subfamilies		GTRPY	Subfamilies
S-1	TRPC	TRPN	S2-1	TRPML	TRPP	S-1	TRPY
S-2	TRPS	TRPM					
S-3	TRP VL	TRPV					
S-4	TRPA	—					

## 2 Role of TRP Channels in Health and Disease Conditions

In the TRP superfamily, nine proposed families are split into two groups with subfamilies (Himmel & Cox, 2020). There is also an additional family labeled as TRPY that is not in either of these two groups (Table 1). Considering group 1 characteristics, each member of this ion channel family is structurally unique and leads to the diversity of functions; however, these ion channels have some commonalities that differentiate this group from group 2 or TRPY.

Considering the second group of these ion channels, the most distinguishable trait of group 2 is their long extracellular span between the S1 and S2 transmembrane segments. Members of these TRP channels are also lacking in ankyrin repeats as well as a TRP domain. In general, TRP channels as plasma membrane machinery play roles in Ca^2+^ and Mg^2+^ transport and modulation of ion entry driving forces. TRPs usually form signaling complexes by interacting with other proteins and work through signaling pathways that are mostly unknown (Zheng, 2013). Noteworthy is that how the sensing domains are coupled to channel gating is not fully understood, but valuable information has been reported. For instance, some research reported that TRPs show modest voltage sensitivity, most likely because of a lack of regularly spaced lysine or arginine residues (gating charges) in their S4 helices structures (Long et al., 2005; Catterall, 2010). Gating charges are a symbol of voltage-gated ion channels and allow the movement of the S4 helix to be consistent with the membrane electric field, which sequentially exerts a force on the S4–S5 linker to eventually open or close the pore (Long et al., 2005; Catterall, 2010; Vargas et al., 2012). It has also been described that voltage sensor-like domains could affect channel gating upon interacting with chemical ligands. Away from the preserved transmembrane core, each TRP subtype is defined by a collection of unique soluble domains that also may contribute to channel assembly and trafficking. These domains can also serve as receptor sites for various endogenous cellular elements or exogenous ligands (Cao, 2020). Apart from all of the above information, previous studies show that, in a normal physiological condition, TRP channel activation is associated with variety of stimuli such as pain, vision, temperature, pressure and taste (Voets et al., 2005; Story, 2006; Sharif-Naeini et al., 2010; Dietrich, 2019; Aroke et al., 2020). Some of the TRP channels, such as those found in animals to sense hot or cold, are assumed to play as the body’s microscopic thermometers (Wetsel, 2011). Some others act as body sensors for volume, stretch, vibration, and osmotic pressure sensors (Lin & Corey, 2005; Mickle et al., 2015; Nesterov et al., 2015; Duitama et al., 2020). These channels allows the movement of cations including Na^+^, K^+^, Mg^2+^, and Ca^2+^ non-selectively (Gees et al., 2010; Nilius & Owsianik, 2011). Under the atypical circumstance, mutations in TRP ion channels have been found to cause neurodegenerative disorders, kidney disorders, and skeletal dysplasia and play important roles in cancer (Hsu et al., 2007; Prevarskaya et al., 2007; Leddy et al., 2014; Kiss et al., 2020; Braidy et al., 2021). As a result, many researchers focused on the clinical significance of TRPs in various disease conditions. So far, it has been found that there are four types of TRPVs (TRPV1, TRPV2, TRPV3, and TRPV4) which are mainly expressed in pain-sensing neurons, afferent nociceptors, and play roles as thermal and chemical stimuli transducers. More specifically, TRPV1, TRPV2, TRPV3, and TRPM8 play roles as thermoreceptors, and TRPV4 and TRPA1 play roles as mechanoreceptors. Targeting these ion channels involved in thermal, chemical, and mechanical sensations may reduce chronic pain (Levine & Alessandri-Haber, 2007). For instance, it has been shown that TRPV1 agonists would potentially inhibit nociception at TRPV1 ion channels overexpressed in pancreatic tissue (Prevarskaya et al., 2007). One of the TRPV1 agonists, known as capsaicin, which is extracted from chili peppers, has been shown to be effective in neuropathic pain relief (Marrone et al., 2017). Additionally, various studies demonstrated that alteration in the expression of TRP proteins such as TRPV1, TRPV6, TRPC1, TRPC6, TRPM4, TRPM5, and TRPM8 leads to tumorigenesis (Prevarskaya et al., 2007; Santoni et al., 2019; Negri et al., 2020).

An example of the changes in TRP expression is the upregulation of TRPV1 and TRPV2 ion channels at Golgi apparatus, endoplasmic reticulum, and surrounding structures in breast cancer patients associated with worse survival (Lozano et al., 2018). In a study performed by Ma and coworkers, it has been shown that TRPV2 play critical roles as an enhancer for H_2_O_2_-induced cytotoxicity by the inhibition of Akt and Nrf_2_ and early activation of p38 and JNK1 together as important implications for inhibition of oxidative adaption as well as drug resistance (Ma et al., 2015). Besides, other studies showed that the TRPM family of ion channels, including TRPM2, TRPM4, and TRPM8, are overexpressed in prostate cancer with more aggressive effects (Wong et al., 2019). TRPM3 ion channel was also found to be a promotional factor for growth and autophagy in renal cell carcinoma (Hall et al., 2014). TRPM4 and TRPM5 also have been shown to be overexpressed and have oncogenic properties in diffuse large B-cell lymphoma and melanoma, respectively, (Palmer et al., 2010; Loo et al., 2017). In addition to the TRPs involved in cancer, other studies reported their function on the neural system as well as sensation.

For instance, in a study performed by Deng and coworkers, it has been shown that the TRPV3 channel as a common ion channel involved in the regulation of crucial neurons in the spinal cord or brain pain pathway (Liu et al., 2011) could interact with S100A4 (Deng et al., 2021). This interaction enhances TRPV3 functions on ion flux regulation (Deng et al., 2021) and links the Ca^2+^ binding protein (S100A4) and the TRPV3 ion channel. Given that, this finding suggests that the TRPV3 regulatory mechanism might have significant therapeutic and diagnostic implications in TRPV3/Ca^2+^-associated diseases (Deng et al., 2021). In a different study performed in animal models, it has been shown that a vesicular trafficking protein known as SNX11 in response to temperature changes modulates mouse behavior (Huang et al., 2020). Accordingly, it has been suggested that this protein may regulate thermal perception by changing the functions of TRPV3 on the plasma membrane of thermally sensitive cells (Huang et al., 2020). Moreover, Hung et al., in other research, revealed that TRPV1, TRPV2, and TRPV4 are functionally expressed in human esophageal squamous cells (ESCC), and the over-activation of TRPV1 and TRPV4 stimulates the proliferation and migration of these cell types. (Huang et al., 2019). Altogether, these studies showed the involvement and importance of the TRP family of ion channels in many different physiological functions.

However, noteworthy is that even though much progress has been made in previous decades for the discovery of TRP families’ roles in health and disease conditions, there are still gaps in our knowledge regarding targeting these ion channels. The subsequent sections of this review provide some information on the group 1 and group 2 members of TRPs and their potential roles as drug targets. Also, there will be a review of the recent findings that used specific drug compounds (agonists, antagonists, or modulators) to target TRP channels. The collected information provides a framework for a better understanding of the physiological significance of drug compounds in regulating TRPs involved in diseases and affects both drug development and clinical indications. For instance, it might help improve our knowledge of treating various types of pain controlled by members of TRPs. Next, we also addressed the challenges facing the study of TRP-targeting compounds, including the need for appropriate screening approaches. As a whole, we will discuss various drug discovery technologies and focus on a fully automated high throughput format of non-radiolabeled tracer-based flux assay coupled with the ion channel reader (ICR) technology for efficient, precise, and reproducible ion channel activity screening. This modern drug discovery technology is responsive to adaptation for studying different ion channels, including the potassium (K^+^), sodium (Na^+^), chloride (Cl^−^), and calcium (Ca^2+^) channels, as well as ion transporters, by using suitable tracer ions.

## 3 TRP Channels as Potential drug Targets

TRP channels (Group 1 and 2 Members), as indicated in Table 1, have a critical role in sensory perception, cellular physiology, and pathology by acting through multiple signaling molecules (Tomilin et al., 2016; Koivisto et al., 2021). These ion channels have several subtypes that could get activated by physical or chemical stimuli (Harteneck et al., 2011). Some of the TRP subtypes act as non-selected cation channels, whereas others regulate the membrane potential through calcium influx (Buijs & McNaughton, 2020). Alteration in the activity of TRP channels could impact cellular functions through modifications in protein synthesis (Perozo et al., 1999). The significance of these channels is well known in several diseases such as pulmonary diseases, cardiac hypertrophy, remodeling, heart failure, obesity, cancer, genito urinal, dermatology, and many more (Banner et al., 2011; Shapovalov et al., 2016). In the following sections, we discussed the involvement of some of these ion channels in diseases and argued their potential as drug targets.

### 3.1 Respiratory Diseases

The sensory nerve in the respiratory system innervates both upper and lower airway tracts, including smooth muscles, larynx, bronchial tree, and lungs. On exposure to any foreign particle, these sensory nerves interact with the central nervous system (CNS) for rapid central reflexes and activation of protective mechanisms, including inflammation, mucus production, and airway constriction (Canning & Spina, 2009). However, these protective mechanisms on prolonged exposure can lead to severe conditions like Asthma and chronic obstructive pulmonary disease known as COPD (Frossard et al., 1989). Increasing evidence has suggested the presence of TRP channels (TRPA1, TRPV1, TRPV4, TRPM8) in epithelial cells, smooth muscles, sensory afferent nerve, immune and inflammatory cells suggesting their potential role as drug targets (Fernandes et al., 2012; Gallo et al., 2017). Modification in the expression of these channels have been reported to inhibit the release of inflammatory markers in asthmatic attack (Chen et al., 2021; Li et al., 2022). Moreover Naumov and coworkers reported the involvement TRPM8 and TRPA1 in the pathogenesis of COPD (Naumov et al., 2021). This suggests that the TRP channels are a potential drug targets.

#### 3.1.1 TRPA1

TRPA1 is an extensively studied TRP channel because of its role in the pulmonary defense system. TRPA1 is a voltage-dependent, calcium-permeable channel discovered in cultured human fibroblasts (Mukhopadhyay et al., 2011); however, now it is identified that it is also present in the sensory vagal neurons, jugular veins, and nodose ganglia (Lin et al., 2020). Exogenous or endogenous irritants can activate these specific channels. Examples of these irritants include mustard oil, cigarette smoke, cinnamaldehyde, scents, reactive oxygen species (ROS), bradykinin and prostaglandins, and many more. (Lin et al., 2015; Taylor-Clark. 2016; Arenas et al., 2017). Activation of the TRPA1 channel on exposure of any irritant leads to a second messenger cascade along with the intracellular movement of Ca^2+^ ions. Several studies have demonstrated the role of Ca^2+^ ion movement through TRPA1 (Zurborg et al., 2007). Experiments also supported the role of TRPA1 antagonists or TRPA1 knockout mice in the treatment of Asthma and bronchitis (Mukhopadhyay et al., 2016; Van den Berg et al, 2021). All in all, the studies mentioned above support the importance of these ion channels and their potential role as drug targets. TRP channels have unique features that allow the incomparable flexibility and critical control of Ca^2+^ ion homeostasis. These channels are mainly involved in regulation of intracellular Ca^2+^ as an essential second messenger and also modulate the different sensory transduction pathways.

#### 3.1.2 TRPV1

Transient receptor potential cation channel subfamily V member 1 (TRPV1 channels, as well known as the capsaicin receptor or vanilloid receptor 1 are present on neurons existing in C- and A-delta fibers primarily identified as nociceptors (Morgan et al., 2019). They cover the entire respiratory tract from the nose to alveoli, smooth muscles, and blood vessels (Xiong et al., 2020). These channels are non-selective cation channels that may be stimulated by a wide range of exogenous and endogenous chemical and physical stimuli. Grace and coworkers observed the interaction between TRPV1 and TRPA1 as the critical regulator of sensory neurons in response to inflammatory discharge (Grace et al., 2012). In addition different study performed by Li and colleagues reported downregulation of TRPV1 expression in association with reduced levels of cytokines and inflammatory markers (Li et al., 2022). Modulation of TRPV1 channels has also been shown to activate or inhibit downstream calcium flux and TCR signaling pathways involved in inflammatory responses (Bertin et al., 2017). These proteins could be promising drug targets considering the expression of the TRPV1 channels and their contribution to the respiratory system.

#### 3.1.3 TRPV4

TRPV4 is a temperature-sensitive non-selective cation channel for calcium and magnesium ions flux (Watanabe et al., 2002). TRPV4 channels are fewer on the lungs’ peripheral sensory neurons than TRPV1 and TRPA1. Its presence has also been reported in rodent sensory, trigeminal, dorsal root, vestibular, nodose, and jugular ganglia (Silverman et al., 2020). These channels are known to cause depolarization of A-delta fibers in guinea pig and human vagus nerves, which TRPV4 and P2X3 antagonists blocked (Mazzone & McGarvey, 2021). In another study, TRPV4 channels affected neurogenic inflammation (Vergnolle et al., 2010). Given the anti-inflammatory potential of the TRPV4 antagonist, these channels could play roles as potential drug targets against inflammatory lung diseases such as Asthma, chronic inflammatory lung disease (COPD), and pulmonary fibrosis.

#### 3.1.4 TRPM8

TRPM8 is a member of the melastatin family. It is present in cold-responsive areas, primarily in vagal afferent neurons within the dorsal root and trigeminal ganglia (Dhaka et al., 2008). TRPM8 mediated current rises with the decreasing temperature and reaches a maximum of 10°C. The activation of vagal afferent neurons is known to be associated with cold-induced respiratory responses via autonomic nerve reflex. Hence activation of TRPM8 channels may cause increased airway resistance through autonomic nerves in response to a cold stimulus. It can also be activated using a cooling agent such as menthol (Andersson et al., 2004). However, contradictory responses have been reported, showing that the inhalation of menthol suppressed citric acid-induced cough response in guinea pigs without activating the TRPM8 channel (Plevkova et al., 2013). Considering TRPM8’s direct association with the respiratory tract, these channels could be considered as prospective drug targets for pulmonary diseases.

### 3.2 Cancer

Despite recent advancements in cancer treatment, the chances of treatment failure, re-occurrence, and mortality are still high. It happens due to the transformation of average cell growth to uncontrolled proliferation and migration. This type of tumorigenic transformation occurs due to mutations in several downstream signaling proteins (Skinner et al., 2012). Among those proteins, TRP channel families have been studied and identified to play a role in specific phases of cancer progression (Yang and Kim, 2020). Several subfamilies of TRP channels are known for transporting calcium, sodium, and magnesium ions. The modulation in the function of the TRP channel subfamily (TRPC, TRPM, TRPV) is associated with the presentation of tumor progression and clinical markers (Gao N. et al., 2020; Zhang L. Y. et al., 2020; Qin et al., 2020). Kaplan-Meier, univariate and multivariate Cox regression showed the link between TRPM8 with pancreatic cancer, TRPM7 with bladder cancer, esophageal squamous cell carcinoma, and TRPV3 with NSCLC (Zhao et al., 2022). Consequently, targeting TRP channels could be an excellent lead in finding the new anticancer drug treatment.

#### 3.2.1 TRPC

Several studies have reported the role of the TRPC subfamily in cancer. In colorectal carcinoma, TRPC1 showed increased CaM-mediated PI3K/Akt signaling proteins responsible for tumor progression (Sun et al., 2021). The pharmacological blockage of the TRPC3 through inhibition and CRISPR mediated TRPC3 knockout (the technology that can be used to edit genes and known as the clustered regularly interspaced short palindromic repeats) helped pause the overgrowth of gastric cancer in both *in vitro* and *in vivo* models (Lin et al., 2021). Moreover, Zhang and colleagues supported the results for TRPC1, where they found TRPC1 mediated EMT (epithelial-mesenchymal transition) exacerbation in gastric cancer (Zhang Z. et al., 2020).

#### 3.2.2 TRPV

Changes in TRPV channel expression are well known to be associated with cancer-related markers. Wang and coworkers reported the presence of TRPV gene alteration as a potential target in 33 cancer types, including breast, ovarian and pancreatic cancer using multi-omics data extracted for the Cancer Genomic Atlas (Wang et al., 2022). Other studies also reported the role of TRPV1 levels in colorectal cancer progression (Jiang et al., 2022), gastric cancer (Liu et al., 2022), and breast carcinoma (Erin et al., 2021).

#### 3.2.3 TRPM

A study of TRPM7 expression using Zinc (Zn) depletion induced MDMX degradation model showed the direct correlation of TRPM7 modulation and increased tumorigenesis (Wang et al., 2021). Fererra and coworkers in a different study also reported the TRPM2 mediated activation of K^+^ channels in cancer cells responsible for metastatic progression of melanoma (Ferrera et al., 2021). The studies mentioned above supported the idea that targeting these channels could be a potential strategy for targeting various types of cancer.

### 3.3 Cardiometabolic Diseases

The heart’s prime and only function is blood circulation to peripheral tissues and organs to meet their metabolic requirements. In a cardiac cycle, most intracellular calcium ions to cause myocardial contraction are from the L-type calcium channel, which is the main route for calcium entry into cardiac myocytes (Bodi et al., 2005). It has been stated that calcium release in cardiomyocytes could be mainly from the sarcoplasmic reticulum (Diaz et al., 2005). Interestingly, later, TRP channels were shown to be associated with calcium regulation in cardiac contraction (Watanabe et al., 2008). The mechanism behind myocardial cells’ regular contraction and relaxation involves inotropic and chronotropic effects (Massion and Balligand, 2003). The changes require the timely movement of calcium ions through channels, including voltage-gated, ligand-mediated, and mechanosensitive calcium channels. Many of these calcium channels are referred to as TRP mediate ion channels (Gao S. et al., 2020; Wen et al., 2020).

An interesting study performed on TRPM4 showed that the deletion of the TRPM4 channel improved cardiac contractility in both normal and MI-exposed mice (Medert et al., 2020). Medert and colleagues, in fact, showed that the attenuation of the type channel’s negative modulator using TRPM4 deletion improved cardiac function (Medert et al., 2020). Moreover, the activation of calcium-mediated signaling pathways is also responsible for cardiac hypertrophy, fibrosis and angiogenesis after the ischemic response (Cheng et al., 2020; Falcon et al., 2020). Captivatingly it has been reported that there is an approximate 50% reduction in left ventricular hypertrophy induced by transverse aortic constriction with selective deletion of the TRPM4 channel (Guo et al., 2021). Dragun and coworkers remarkably reported the controversy in the expression of TRP subfamily proteins in heart failure. Their study showed increased expression of TRPC1, TRPC5, TRPM7; however, decreased expression of TRPC4 and TRPV2 in the diagnosis of end-stage heart failure (Dragun et al., 2019). This discrepancy in the activity of TRP channels is also present in metabolic diseases. Though inhibition of the TRP channels is beneficial in cardiac diseases, its role in diabetes is the opposite. Activating TRP channels in Beta-islet cells in the pancreas causes depolarization and insulin release. However, its inhibition could decrease the release of insulin.

Moreover, TRP channels being cationic channels, promote the glucose-stimulated insulin secretion from beta islet cells. In the process, the entry of glucose and activation of TRP channels causes depolarization of the cell and insulin release (Yamada et al., 2016). Similarly, Funazaki et al. also showed that the supporting activation of TRPM2 by Imeglimin enhanced the release of insulin secretion and showed it to be a possible tool for type 2 diabetes (Funazaki et al., 2022). Also, there are several other forms of TRP channels such as TRPC, TRPM, TRPV, and TRPML in blood vessels, endothelial cells, vascular smooth muscles, and perivascular adipose tissue. All these subforms of TRP receptors are involved in vascular alteration in cases of diabetes and obesity (Moraes et al., 2021). Hence, targeting TRP receptors could be considered as potential drug targets and have beneficial effects in cardiometabolic diseases.

## 4 Drugs (Compounds, Agonists, and Antagonists) For Targeting TRP Ion Channels

After discovering TRP channels and finding their role in different diseases, several ligands have been introduced that act as agonists or antagonists, and a few of them showed therapeutic potential. For instance, capsazepine and 4-hydroxy-5-iodo-3-methoxyphenylacetate antagonized TRPV1 channels, reducing the capsaicin-induced paw flinching in rats (Seabrook et al., 2002). Similarly, Yohimbine presents analgesic properties by inhibiting vanilloid receptors (VR1) in *in-vivo* experiments (Dessaint et al., 2004). Carvacrol has been shown capable of modulating cardiac electrical activity through its inhibitory potential on TRPM7 channels at both cardiac and vascular levels. These changes were reported without impacting resting membrane potential and can have therapeutically potential in cardiac diseases (Almanaityte et al., 2020). In other studies, Majeed et al. reported the controversial role of rosiglitazone in the modulation of TRP channels. Rosiglitazone inhibited the activation of TRPM2 and TRPM3, whereas it promoted the stimulation of TRPM5. Troglitazone and pioglitazone also showed similar results in inhibiting TRPM2 and TRPM3, without any effect of TRPM8 channels (Majeed et al., 2011).

Nonetheless, several other compounds were tested at both preclinical and clinical levels for their effect on the modulation of TRP channels. These compounds were considered essential and assessed at different clinical trial levels against several diseases. Here we demonstrated some of the compounds under clinical trials that are or were used for targeting the TRP ion channels (Table 2).

**TABLE 2:** Compounds under clinical trials to target the transient receptor potential channels (TRP ion channels). The information was retrieved from the ClinicalTrials.gov database.

Channel	Compound	Condition	MOA	Primary outcome	Status	Clinical trial Number
TRPV1	SB-705498	Migraine	Antagonist	Pain-free at 2 h post-dose	Phase II completed	NCT00269022
	SB-705498	Dental pain	Antagonist	Reduction in pain intensity using Visual Analogue Scale	Phase II completed	NCT00281684
	SB-705498	Rhinitis	Antagonist	Mean total symptom score elicited by 1-h cold air challenge	Phase II completed	NCT01424514
	NEO6860	Osteoarthritis	Antagonist	Safety and tolerability of NEO6860	Phase I completed	NCT02337543
	PAC-14028 cream 1.0%	Atopic Dermatitis	Antagonist	Percent of patients with IGA score of 0 (clear) or 1 (almost clear)	Phase III completed	NCT02965118
	XEN-D0501	Chronic Idiopathic Cough	Antagonist	Reduction in daytime cough frequency	Phase II completed	NCT02233699
TRPV2	Probenecid	Fontan	Agonist	Improvement in End Diastolic and End Systolic Volume	Phase IV completed	NCT03965351
TRPV6	SOR-C13	Cancer	Antagonist	Toxicity	Phase I completed	NCT01578564
TRPM8	Methoxypropanediol	Non Randomized parallel	Atopic Dermatitis	Antagonist	Mitigation of pruritus markers	NA
TRPC5	GFB-887	Randomized parallel	Diabetic nephropathy	Antagonist	Percentage change in Urine Protein-to-Creatinine Ratio (UPCR)	Phase II recruiting

## 5 Current Technologies in the Study of Ion Channels

In recent years, a number of techniques have emerged to discover ion channels and their activities. Three main techniques that are well-known for studying ion channels’ pharmacology include the 1) ligand-binding assay, 2) membrane potential measurement, and 3) ion flux assay **(** Yu et al., 2016). The selection of techniques is dependent on the type and quality of ion channel protein optimized for a particular experiment. For instance, the ligand binding assays can assess the affinity of a molecule to bind the ion channel but not the movement of an ion across the channel, hence referred to as a non-functional assay. In contrast, the ion flux assay and membrane potential assay define the movement of ions through the channel and are considered functional assays. Apart from these, patch-clamp as a gold standard electrophysiological technique used to assess ion channel behavior can be used to study ion channels in neurons, cardiomyocytes, oocytes, and muscle fibers (Kornreich, 2007). In patch-clamp experiment, microelectrodes create suction and form a firm seal with the cell membrane by isolating a patch containing ion channel of interest. This clamp measures the voltage difference and/or amount of current that flows across the cell membrane (Hill & Stephens, 2021). The technique is versatile and can be used to study ion channel behavior in the presence of drugs, ions and other analytes.

Regardless of the assay types, the instruments used for the readouts are four different types, including the radiometric, fluorometric, electric, and spectrometric (Yu et al., 2016). These techniques are based on high throughput screening, and each of them has a few pros and cons depending on the type of experiment. Here we tried to briefly review these techniques and further elaborate on their application for TRP ion channels drug discovery.

### 5.1 Ligand Binding Assay

The ligand-binding assay investigates the binding or detachment of drug molecules to the ion channel. However, it is limited to the detection of specific ligand binding sites. In other words, this method incorporates a radiolabeled ligand binding assay, in which the binding of a radiolabeled molecule to the ion channel is recoded without any information regarding channel function (Hulme and Trevethick, 2010). In addition to the described binding method, there is also a surface plasmon resonance method. The targeted ion channel protein is stabilized onto the metal surface, and the ligands pass through the metal surface as a mobile phase. A fraction of light (incident light) links with the delocalized electrons in the metal film during this procedure (Huber et al., 2017). Upon binding the ligand with an ion channel, the intensity of the reflected light changes and will be measured by the instrument. Both methods cannot assess whether ligand attachment opened (agonist) or blocked (antagonist) the channel. Hence, they are not used frequently these days.

### 5.2 Membrane Potential Assay

The electric potential is a biophysical variable that exists because of potential differences across the cell’s plasma membrane. The presence of exogenous or endogenous stimuli leads to the activation of ion channels and an electrochemical gradient (Subramanyam & Colecraft, 2015). These stimuli can be internal or external exposure, such as drugs. The changes in ionic concentration depend on the opening and closing of the ion channel. Based on these electrochemical differences, we can assess the effect of a drug on the activity of the ion channel. The depolarization of the cell includes the opening and closing of several channels at the same time. Hence, it is challenging to assess the activity of only one channel when the gradient can be changed with other channels. This is the main disadvantage of this technique. However, advances have been made in which the voltage-sensitive dyes are used to measure potential difference, and the technique is called fluorescence resonance energy transfer (FRET) (Sekar & Periasamy, 2003). In this technique, two probes are inserted in the cell membrane, and a give and take relationship is created between fixed donor fluorophore and voltage sensing dye. The movement of ions through the membrane of the cells changes the entire fluorescence emission influence. Though this technique has been used for years, the sensitivity is not good because of insensitive nonlinear readouts.

### 5.3 Ion Flux Assay

Labeled tracer ions are used in this technique to measure the movement of ionic flux across the cell. The channel opens through a stimulus that allows the movement of ions through the channel across the cell membrane (Gill et al., 2003). The technique is based on functional changes in the activity of the ion channel. Depending on the type of trace element labeling, the detection can be done by radioactive decay, fluorescent, spectroscopy, or electronic plate readers. Radioisotopes tracers are used in the radioactive decay method, such as 22Na^+^ for Na^+^, 45Ca^2+^ for Ca^2+^, and 86Rb^+^ for K^+^ channels for cellular influx or efflux desired ions (Nimigean et al., 2006; Roberts et al., 2016). For instance, the most commonly used experiment involves the use of 86Rb^+^ ion for the detection of potassium and non-selective cation channels (Parihar et al., 2003). The cells carrying channels of interest are incubated with radiolabeled Rb^+^ ion. After the desired incubation, the cells are washed and initiated with potassium chloride as it hyperpolarizes the cell and allows the efflux of Rb^+^. The effect of an agonist or antagonist can be measured by adding it to the open ion channel. The movement of radioactive Rb^+^ tracers quantifies the functional changes in the ion channel due to agonistic or antagonistic effects (Cheng et al., 2002). Though the technique involves assessing the functional activity of ion channels, it is complicated to deal with radioactive waste material.

Further, to improve the throughput of the experiments and fulfill the gaps, a fluorescent-based ion flux assay was developed. This assay uses ion-specific dye, which alters the fluorescence emission upon a change in the concentration of that ion across the cell membrane. Though the assay detects the functional changes in the ion channel, it is limited to the availability of ion-specific dyes. Notably, the fluorescent-based ion flux assay, the most common HTS method today, such as Fluorometric Imaging Plate Reader (FLIPR), has significant drawbacks. Direct detection of endogenous ions, coupled with complex mechanisms of ion concentration changes, leads to false positives easily. For example, the release of internal calcium stores upregulates intracellular free calcium concentration. Specific ionic fluorescent dyes are expensive and unsuitable for large-scale compound screening, and fluorescence detection is prone to light interference and has low resolution. Calcium sensing dye is the most commonly available dye; therefore, this assay can be used to assess Ca^2+^ channel activity (Russell, 2011). After that, a voltage-clamp method was introduced, which detects the electronic negative feedback circuit from activated cells and measures the changes in membrane potential due to ionic movement (Hernandez-Ochoa & Schneider, 2012).

Apart from the aforementioned techniques, new technologies have been developed to increase the throughput and specificity for a particular ion channel with reduced noise in the recordings. These emerging technologies include automated electrophysiology, automated whole-cell patch-clamp, automated voltage clamp of Xenopus oocytes (Papke & Smith-Maxwell. 2009). These techniques might help to find the reason behind the increased or decreased expression of the ion channels, either pathologically or physiologically. One of such techniques is the Aurora Biomed initiated a non-radiolabeled Rb assay that can assess the functional changes through a spectroscopic approach to deal with the drawback of the radioactive ion flux method as well as the fluorescent-based ion flux assay. Aurora’s team validated these assays for K^+^, Cl^−^, Na^+^, as well as Ca^2+^ channels along with the activity of Na^+^/K^+^-ATPase (Qi et al., 2014). In the subsequent sections of this review, we will discuss in more detail how the ICR instrument using the principles of flux assay would be an excellent choice to detect the ion channel activity of TRP channels.

## 6 Technologies For the Study of TRP Ion Channels

As discussed in previous sections, several techniques were used to discover the TRP family of ion channels (Table 3). In the era of TRP as an emerging target for drug discovery against many life-threatening diseases, thorough screening for the activity of these channels is significantly required. TRP ion channels are known for conducting cations (Ca^2+^ or Mg^2+^) across the cell membrane and are responsible for physiological and pathological processes. Hence, many studies have reported using calcium-labeled fluorescent dye to assess the activity of TRP subfamily channels. For instance Song and her coworkers used a novel suspension B lymphocyte cell line that expresses TRPM2 to create a cell-based assay, which monitored TRPM2-dependent Ca^2+^ transients by utilizing a Ca^2+^-sensitive fluorescent dye, Fluo-4 NW (Song et al., 2008). Additionally in a distinct study, Sanchez and colleagues developed a protocol that used Fluo-4 NW Ca^2+^ assay to screen thermoTRP compound modulators for TRPV1 and TRPM8 (Cordero-Sanchez et al., 2019). Besides to the Ca^2+^-sensitive fluorescent dye assay, Castillo et al. developed a fluorescent dye-based bioassay to study TRPM7-mediated Mn^2+^ influx in HEK293 cells (Castillo et al., 2010). However it is of note that assays using fluorescent dyes as tracers for investigation of ion channel activities, have some weaknesses and are largely limited to the availability of high performance ions-pecific indicator dyes (Dai et al., 2008; Song et al., 2008; Miller et al., 2011). Due to the drawbacks of this method in the following section, we discussed the application of Ion channel technology (ICR) based on a fluxed assay to study TRP Channels.

**TABLE 3:** A list of studies reporting the TRP ion channel drug discovery using techniques other than Ion Channel Reader (ICR) technology.

TRP Ion Channel	Technique used	Model	Drug, agonist antagonist	Reference
TRPM2	Ca^2+^-sensitive fluorescence dye, Fluo-4 NW	Novel suspension B lymphocyte cell line	TRP channel modulator	Song et al. (2008)
TRPC4	Whole-cell patch-clamp	HEK293 cells	ML204, antagonist	Miller et al. (2011)
TRPM8	QPatch HT	Menthol activated ion channel	Antagonist	Beck et al. (2010)
TRPM7	Mn^2+^ sensitive fluorescent dye	HEK293 cells	Antagonist	Castillo et al. (2010)
TRPV1 and TRPM8	Ca^2+^-sensitive fluorescence dye, Fluo-4 NW	Puro MEM culture media	Antagonist	Cordero-Sanchez et al. (2019)
TRPM8	Radiometric Calcium imaging and electrophysiology using FLIPR	cold, voltage, and phosphatidylinositol-4,5-bisphosphate activated ion channel	Agonist	Bandell et al. (2006)
TRPV1	Automated patch clamp	TRPV1-null mice	Antagonist	Zicha et al. (2013)

## 7 Application of Ion Channel Technology For the Study of TRP Channels

While there is increasing information on the role of TRP ion channels in health and disease conditions, little is known about the compounds and peptides that can be used as modulators of these channels. The present review in previous sections summarized the available information on the TRP channels and the existing methods and technologies for studying different ion channel families, including the TRPs. As part of the discussion, we would like to elaborate more on the Ion channel reader (ICR) technology and its application to study TRP channels. For the study of TRPs, similar to other ion channels, patch clamping is the gold standard electrophysiology method. The automated whole-cell patch-clamp assay has the characteristics of the high cost of instruments and consumables, high requirements for cell status, and high technical requirements for experimenters. In previous research studies conducted using ion channel technology for various ion channels and transporters, the ICR high throughput (HTS) technology was validated using flux assays. ICR has been found to be a robust, sensitive, and cost-effective high throughput system for studying various ion channels and transporters. Comparing ion channel reader with high throughput automated patch-clamp technique, ICR is more cost-effective and powerful. For instance, ICR 12000 can analyze up to 60,000 samples per day, whereas automated patch clamps can read less number of samples per day (<20,000). The cost for an automated patch clamp has been reported around €0.10-€0.15 per data point (Bell & Dallas, 2017). However, ICR has a lower cost per data point reading. ICR is compatible with both 96 and 384-well microplates and, compared to high throughput automated patch-clamp and FLIPR, has a higher throughput at a lower cost. Though, it is of note that ICR has a limitation in the number of samples to be read simultaneously, and it can cover up to 12 samples per run. Enabled with an enhanced automated liquid handling and multi-channel sampling feature, ICR is designed for ultra-high throughput ion channel screening of compound libraries with high precision and sensitivity. One of the ion channels’ families widely investigated by ICR and flux assay is the K^+^ channel (Sorota et al., 2005; Chaudhary et al., 2006; Terstappen, 2006; Karczewski et al., 2009; Terstappen, 2011; Na et al., 2020). Some of these families reported voltage-gated potassium channels, including hERG, Kv1.1, Kv1.4, and Kv1.5 stretch-activated potassium channels. Additionally, more studies investigated voltage-gated sodium channels using ICR, including, Nav1.2, Nav1.5, and Nav1.7, and ligand-gated channels including GABAA, P2X, KATP SKCa, BKCa, and nAChR. Moreover, some of the transporters, such as Na^+^/K^+^-ATPase and the Cation-Cl Co-Transporter (CCC), also have been reported to be measurable by ICR. Various publications reported the application of Non-radioactive Rb^+^ efflux to measure hERG blockage (Sorota et al., 2005; Chaudhary et al., 2006; Karczewski et al., 2009). A comparison of efflux assay and data collected using manual patch clamp on hERG channels as one of the key ion channel targets also have been investigated using ICR. The results clearly show that the measurement of Rb^+^ efflux is a reliable and accurate method for predicting the potency of compounds (IC50 values) blocking the hERG channels. The results from these studies are consistent, reproducible, and can be used to measure channel modulation of drugs from several therapeutic areas, including TRP ion channels. Previously, based on sequence similarity and using the information collected from the low-resolution cryo-electron microscopy (EM) structures, all of the members of the TRP channels were expected to look similar to the voltage-gated potassium channels, having four subunits adjacent to a centrally located ion permeation pore (Madej & Ziegler, 2018). Zicha and coworkers using Aurora’s ICR8000 single-channel atomic absorption flame spectrometer determined the concentration of rubidium present in cells (Zicha et al., 2013). Advances in technologies helped improve our knowledge in this regard. Considering the functionality of TRP ion channels, an increase in intracellular Ca^2+^ concentration in response to stimuli is a good indicator of these channels’ functional expression (Zheng, 2013). Calcium channels control not only disease-related functions but also important physiological functions throughout the body that might be perilous if interrupted (McDonough, 2013). Many of these channels have unknown mechanisms of action. It is also hard to find appropriate lead compounds for these channels due to the difficulty of screening new compounds. As a result, calcium channels are a challenging area of drug development, and ICR could be a potential approach for targeting this family of ion channels.In general, TRP channels as plasma membrane machinery systems play roles in Ca^2+^ and Mg^2+^ transport and modulation of ion entry driving forces. TRPs usually form signaling complexes by interacting with other proteins and work through signaling pathways that are mostly unknown (Winston & Lutz, 1988). Regarding applying ICR technology and flux assays for TRPs, Aurora Biomed-Discovery performed experiments that showed that ICR is applicable to studying the TRPC3 channels. Interestingly it has been found that the TRPC3 channel involved Ca^2+^ signaling is activated by ATP as an agonist, and as a result, various compounds have been tested to determine their potencies. Results showed that the potency rank order of some antagonists is quite close to the potency order presented by patch-clamp in the literature. The Z′ factor value is significantly conducive for the assay’s QC; as a result, it was suggested that the Li influx assay might apply to other members of the TRP family. As TRP channels have a relatively non-selective permeability to cations, containing calcium, sodium, and magnesium, and ICR has been shown to be a successful assessment tool for both voltage-gated (such as hERG, KCNQ, Kv1.1, 1.4, 1.5, 2P, BK/SK, etc.) and ligand-gated (nAChR, KATP, etc.) ion channels, plus ion pumps and transporters (e.g., Na^+^/K^+^-ATPase). ICR indeed allows researchers also to accelerate their TRP drug discovery and development processes. ICR series of instruments detect various ion types’ movements across membrane proteins by measuring the intracellular and extracellular ion concentrations of interest *via* atomic absorption spectroscopy (AAS). Ion Channel Reader (ICR) employs flux assay technology for ion channel screening by using the non-radioactive ions as the tracers for definite ion channel types. In this technology, the tracer ions are loaded into the cells or the extracellular solution, and then the cells are bathed in a different solution containing the compound/s of interest. This helps to determine the activity of compound/s of interest on the activity of the channels. In more detail, using this system, the number of tracer ions present in the intra and extracellular compartments of the cells is measured using the Aurora Biomed’s sensitive, robust, and high-throughput patented atomic absorbance-based Ion Channel Reader (ICR) instrument. The tracers that are used for studying the activity of different ion channels and transporters include Potassium (K^+^): Rubidium (Rb^+^); Sodium (Na^+^): Lithium (Li^+^); Chloride (Cl^−^): silver (Ag^+^) ions.

For instance, to measure free Ag^+^, an identified silver (Ag^+^) concentration is used to precipitate Cl^−^ ion from the samples as AgCl. Regarding the other ion channels, such as the Calcium ion channels, Ca^2+^ or Strontium (Sr^2+^) are used. For the Na^+^, K^+^-ATPase, the Rb^+^ for K^+^ ions are used. For the K^+^, Cl-co-transporter, the Ag^+^ precipitation for Cl^−^ ion are used. This technique is independent of and corresponds to approaches that rely on voltage operation. Since ion flux is a straight measure of ion channel and transporter activity, these assays are robust and less delicate to turbulences. They are widely used by various pharmaceutical companies and academic research groups. Data generated by the ion channel series using the flux assay are very consistent and predictive of drug potency and help eliminate the bottlenecks in which HTS requires high sensitivity, small sample amount, and quick results.

Flux assays involve about 1.38 × 10ˆ5 cells/well and, as a result, provide multicellular response compared to other techniques quantifying only a small number of ion channels. The ion channels activity measurements made using ICR are very sensitive, with an exceptionally low percentage of CVs among the replicates. The volume of the samples used in the assays is less than 100 μl, which minimizes the amount of testing compound/s used. The IC50 values obtained using ICR were found to be consistent with the results obtained from electrophysiology studies and provide similar rank order for various test compounds. The screenings are carried out in the presence of 1% Dimethylsulfoxide (DMSO), minimizing compound solubility issues. Z′ values of the screens are at all times higher than 0.8, representing high robustness in addition to low variability. Considering a large detection window presented by the screens, test compounds appear as either clear blockers/non-blockers or activators/non-activators. Drug potencies (IC50 values) determined by flux screens are highly correlated with those of electrophysiology. All in all, using the ion channel reader (ICR) instrument series combined with flux assays, the primary screening against ion channel targets or secondary screening to access drug safety concerns is possible.

## 8 Conclusion

There is considerable interest in ion channel research groups and pharmaceutical companies in the TRP family of proteins. This is because almost every cell in the body expresses at least one type of the TRP channel family with different biophysical, mechanical, and pharmacological characteristics. These ion channels are sophisticated signaling machines that sense diverse physiological and environmental signals. Despite the broad range of research done on TRPs, this family of proteins remains an exciting and potentially worthwhile group of drug targets for a wide range of diseases. Among the whole family of TRP channels, those involved in sensory signaling are possibly best characterized and have been used as experimental models for understanding functional aspects of TRPs. Regarding the compounds (either natural or synthetic) current TRP channel-modulators could be studied by various methods in which application of ion channel reader using non-radioactive flux assay could be an asset. The findings demonstrate that the proper target selection and studies related to drugs biosafety help define clinically expressive doses and find ideal therapeutic windows. The overall outcome provides strong support for the importance of TRP ion channels as important drug targets and Flux assay as an excellent primary screening method for these targets, determining drug potencies or as an influential secondary screen for drug safety analysis. This review highlights the importance of improving flux HTS automated technologies using the accurate, reliable, and cost-effective ion channel reader (ICR) instrument for TRP-related research and clinical studies. It also provides a better framework for the application of ICR as a reliable instrument for evaluating natural or synthetic pharmacological agents for treating ion channel–associated disorders.
